# Diel Versus Seasonal Butterfly Community Partitioning in a Hyperdiverse Tropical Rainforest

**DOI:** 10.3390/insects16121247

**Published:** 2025-12-10

**Authors:** Sebastián Mena, Janeth Rentería, María F. Checa

**Affiliations:** 1 WasiLab—Laboratorio de Estudios en Sostenibilidad, Pontificia Universidad Católica del Ecuador, Quito 170525, Ecuador; 2 Museo de Zoología QCAZ Invertebrados, Escuela de Ciencias Biológicas, Pontificia Universidad Católica del Ecuador, Quito 170525, Ecuador

**Keywords:** Amazonia, Ecuador, forest dynamics plot, Lepidoptera, niche theory, Yasuní National Park

## Abstract

Ecological theory suggests that interspecific interactions and environmental heterogeneity promote temporal niche partitioning, whereby species segregate their activity along diel and seasonal axes. For ectotherms, temperature is a critical niche dimension because heat availability regulates activity and phenology. Here, we used data from a hyperdiverse rainforest in the Ecuadorian Amazon to compare community dynamics across two temporal scales and to test their relationship with temperature fluctuations. Butterflies were periodically sampled using Pollard walks in a permanent plot over eight field campaigns spanning two years. We compared environmental temperature fluctuations, diversity metrics, and niche-overlap estimates of community assemblages at both diel and seasonal scales. We recorded 1003 individuals representing 222 species. Temperature differences among seasons were comparable to those observed across times of day. Consistently, our analyses revealed distinct community assemblages across both diel and seasonal scales. Furthermore, butterfly activity tended to increase during warmer hours and in warmer seasons, yet overlap in activity within these timeframes was low at both the species and subfamily levels. These results highlight the contribution of both abiotic drivers and biotic interactions in structuring butterfly temporal abundance. More broadly, our study underscores the importance of explicitly considering temporal dimensions when examining tropical biodiversity.

## Simple Summary

Tropical forests contain the vast majority of butterfly species worldwide, but the reasons why so many species coexist in the same area remain unclear. One explanation is that species partition their activity across time, reducing direct competition for resources. However, since butterflies rely on environmental heat to fly, feed, and reproduce, temperature is expected to strongly influence when different species are active. In this study, we monitored butterflies over two years of repeated sampling in the most biodiverse site documented to date, located in the Ecuadorian Amazon. We recorded more than one thousand individuals from 222 species. Our results show that differences in temperature between morning, midday, and afternoon were as pronounced as those observed across different seasons. Butterfly assemblages changed significantly between times of the day and between seasons, and activity was generally higher under warmer conditions. However, species and subfamilies showed little overlap in their activity periods, suggesting that temporal separation is an important mechanism that allows many species to coexist. These findings reveal how both daily and seasonal temperature variation contribute to structuring butterfly communities, with broader implications for understanding biodiversity and its vulnerability to climate change.

## 1. Introduction

Tropical rainforests are the most biodiverse places on Earth. They account for nearly half of the world’s forest ecosystems, all in 7–10% of the Earth’s land area [1], which harbors half of the world’s species diversity [2], including 90% of all butterfly species [3]. Documenting patterns to disentangle the mechanisms that support such hyperdiversity is a major goal of tropical community ecologists and evolutionary biologists [4]. Ecological theory suggests that interspecific interactions and environmental heterogeneity jointly shape species coexistence by promoting niche differentiation [5]. Uncovering the spatial and temporal dimensions of these mechanisms is therefore essential for understanding how such extraordinary levels of biodiversity are structured and maintained.

Temporal niche partitioning—species activity segregation along seasonal or diel axes—is considered one key mechanism by which species avoid competitive exclusion and coexist within the same spatial habitat [6]. For tropical rainforest butterflies, temporal scales have been acknowledged as a key niche dimension [7]. Butterfly temporal abundance fluctuations have been reported to show annual [8,9], bi-annual [10,11], and multi-annual [12] patterns; with strong peaks in abundance associated with increased resource availability [11], timing of host plant flushing and flowering [13], or climatic anomalies such as flooding events [14]. Likewise, studies have found shifting patterns of diel activity, with butterflies being active throughout the whole day, having activity peaks at midday or appearing the most during the early morning and/or late afternoon [15,16]; and behaviors such as feeding, courtship, migration and oviposition have been reported as separated among particular hours of the day [17,18]. Indeed, while temporal variability changes the physical and chemical environment within which organisms live and interact [19], moving among favorable locations in a temporally varying environmental palette domain allows species to stitch together viable niches [20]. Exploiting different temporal windows may minimize overlap in resource use and alleviate competitive pressure, thus promoting coexistence within a shared spatial environment [21].

Niche-based community assembly theory suggests that species coexistence is facilitated by environmental heterogeneity and the evolution of niche segregation in response to interspecific interactions [6,7,8,9,10,11,12,13,14,15,16,17,18,19,20,21]. Environmental heterogeneity is suggested to maintain diversity via three main mechanisms: (i) different resources and conditions, creating more available niches; (ii) more shelters and refuges, which promote species persistence; and (iii) fostering population isolation along environmental gradients, promoting divergence and eventual speciation. [22]. Species interactions are suggested to sustain diversity via two main mechanisms: (i) Reduced competition by negative frequency dependence, where abundant species deplete resources quicker; and (ii) predator-mediated coexistence, where predators suppress dominant competitors, freeing niche space for subordinate species [23,24]. Many studies have acknowledged climate as a major driver of temporal variation in butterfly assemblages. For example, differences in temperature and light availability are correlated with shifts in diversity patterns [25,26], and their influence on butterfly physiology has driven the evolution of functionally diverse flight morphologies [27,28]. Other studies have also highlighted the role of biotic interactions in structuring butterfly assemblages. For example, studies have shown that nectar-feeding butterflies may face competition for nectar [29].

**Figure 1:**
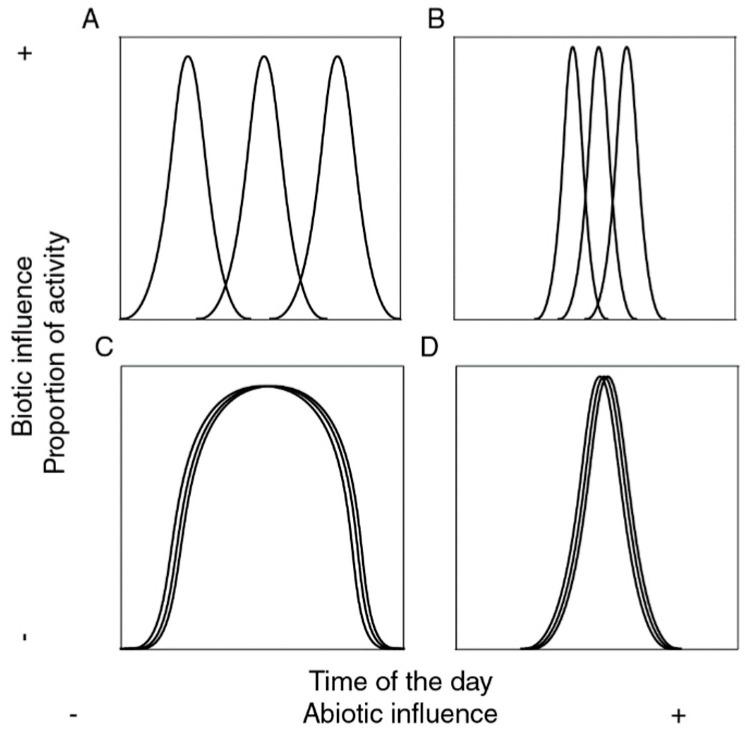
Possible relations between time of the day and species activity in a given community (each line representing a species): (**A**) There is full activity throughout time, but species do not overlap (widespread and segregated); (**B**) Activity happens only part of the time, species do not overlap (narrow and segregated); (**C**) All species are equally active throughout time (widespread and aggregated); (**D**) Species are active only part of the time, their activity overlaps (narrow and aggregated).

Since the partitioning of temporal niches involves a complex interplay between abiotic filtering and biotic interactions, four contrasting patterns have been proposed to address the relative contribution of biotic and abiotic factors structuring them (Figure 1). Thus, a community may be described as a set of species that may be able (or not) to adapt to all ranges of abiotic conditions, and that may be able (or not) to overlap in the use of such conditions. When abiotic factors dominate, species distributions and activity patterns are largely determined by physiological tolerances and environmental filtering, leading to predictable temporal assemblages aligned with climatic cycles [30,31]. In contrast, when biotic interactions such as competition, predation, or facilitation prevail, species temporal segregation emerges even under similar environmental conditions, as species minimize niche overlap to reduce competitive pressure [32]. Intermediate scenarios reflect a continuum between strong environmental filtering and strong biotic structuring, where temporal niche partitioning is shaped by both environmental constraints and interspecific interactions. This framework provides a useful lens to interpret patterns of temporal community assembly across different ecological contexts.

Although days in the rainforests may experience similar fluctuations in climate variables than those across higher timescales or vertical strata, whether there are differences in butterfly assemblages at different times of the day, and whether such differences are comparable to other scales, has been poorly investigated, even in large studies that aimed to cover several spatio-temporal dimensions, e.g., [33]. Here, we examined diel and seasonal dynamics of butterfly communities in Yasuní National Park (YNP), one of the world’s most biodiverse sites [34]. We hypothesized that, consistent with niche theory, YNP butterfly species have occupied available diurnal and seasonal niches and partition their activity throughout the season and the day, and thus exhibit a temporal pattern consisting of stable richness while allowing compositional turnover through time. From this, we predicted that: (1) differences in community structure across months will be comparable in magnitude to those across hours of the day; (2) across hours of the day, (a) community structure and composition will differ significantly, (b) species richness will remain stable, and (c) abundance will be relatively evenly distributed; and (3) biotic factors will contribute more strongly to explaining temporal differences than abiotic interactions.

## 2. Materials and Methods

### 2.1. Study Area

**Figure 2:**
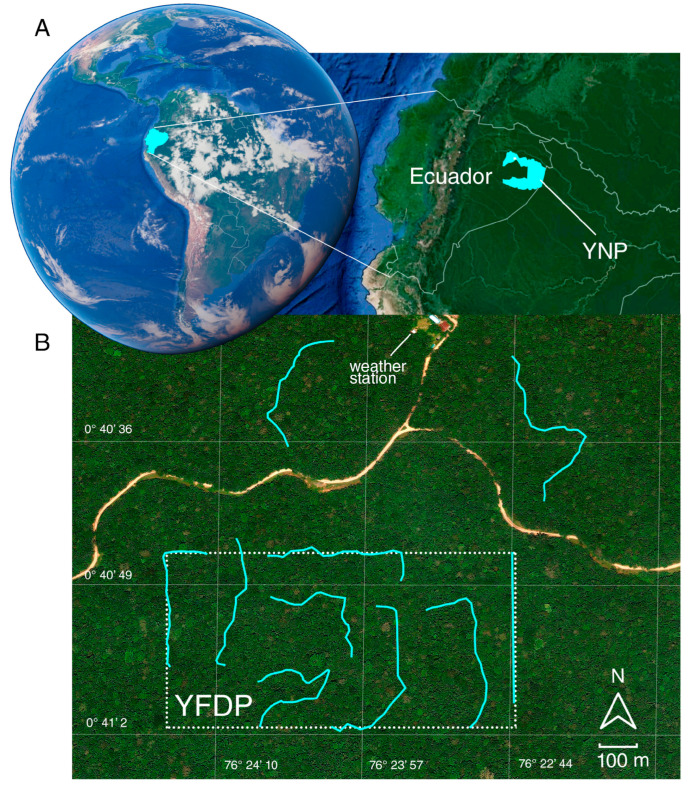
Study area: (**A**) location of Ecuador within South America, the Yasuní National Park (YNP); and (**B**) the 50h Yasuní Forest Dynamics Plot (YPDF). Transects are highlighted in cyan. All images were obtained from Google Earth Pro (v. 7.3, copyright © 2025, Google Earth and Maxar Technologies, Mountain View, CA, USA).

Our study was conducted in the Yasuní Forest Dynamics Plot (YFDP), in Yasuní National Park (YNP), Ecuadorian Amazon (76°24′ W; 00°40′ S) (Figure 2). Compared to other monitoring plots on Earth, the YFDP is exceptionally diverse: 670 tree species have been recorded in 1 ha; 173 and 502 more species than what can be found in the same area in Pasoh, Malaysia and BCI, Panama, respectively [35]. YNP is an evergreen lowland wet forest ranging in altitude from 200 to 300 m.a.s.l. It has a 15–30 m canopy with some emergent trees reaching 50 m [36]. YNP and the adjacent Waorani Indigenous Territory cover 1.6 million ha of forest and comprise the largest protected area in Amazonian Ecuador (~18% of the Ecuadorian Territory) [37]. Because of the convergence of peaks in species richness for key taxa (birds, plants, amphibians, and mammals), YNP is considered the most biodiverse site on Earth [34].

### 2.2. Weather Data

**Figure 3:**
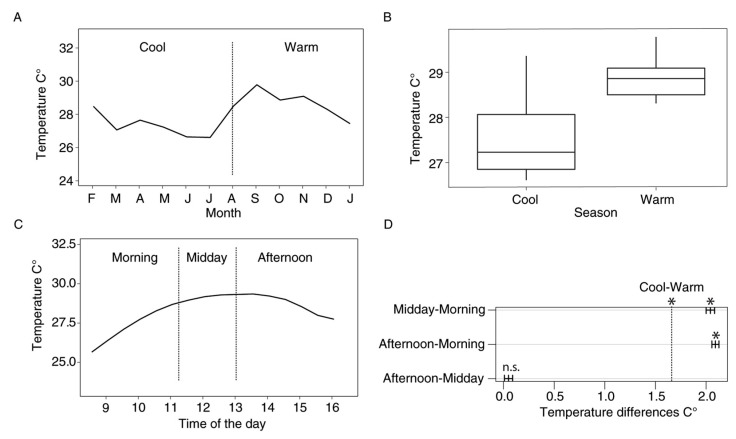
Yearly and daily temperature variability (**A**,**C**) and mean temperature differences (**B**,**D**) in our study site. Significant differences between intervals are marked with an asterisk. Data from years 2015, 2016 and 2018.

To test our hypothesis that diel variations in conditions are similar to those in larger timescales, we used temperature as a proxy for general environmental variability due to its correlation to rain, humidity, and soil moisture [8,38]. Previous studies have identified temperature as an important environmental factor influencing butterfly activities and resource availability [7,39,40,41]. In YNP, temperature has been reported as the most significant climatic factor explaining differences in butterfly richness and abundance throughout the year [8]. Due to its relevance for insect development, metabolic rate, and flight performance [3,42], temperature is a powerful abiotic factor. Therefore, we asked whether temperature variation within times of the day is similar to variation within seasons of the year. For this, we used measurements of air temperature from Estación Científica Yasuní (ECY) weather station [43] from 2015, 2016 and 2018. Our data showed a clear pattern consisting of a significantly higher temperature towards the second half of the year (i.e., the “warm” season. Figure 3A,B), although some buffering of these measurements should be expected to happen inside the forest understory.

### 2.3. Sampling

We used Pollard walks [41], following the standardized protocol from [44]. Pollard walks are a relatively novel approach to butterfly monitoring in the neotropics allowing capturing butterflies of diverse feeding guilds, compared to the traditional Van Someren-Rydon trapping which allows the capture of only either carrion or fruit-feeding butterflies. Moreover, this method permits collectors to have accurate records of species’ time of occurrence during the day [39]. Ten transects were established across the study area—eight within the trail system of the 50-ha YFDP and two in adjacent forest areas. All transects followed ~3 m-wide trails maintained by ECY personnel, running through primary rainforest understory with occasional natural gaps caused by treefalls and slight topographic variation (~50 m). Each transect was located at least 100 m from forest edges associated with the nearby road. Each transect measured 500 m long and the separation between transects was at least 100 m (Figure 2B). Eight sampling periods were conducted, distributed at three-month intervals between July 2015 and May 2016, and between January and October 2018. to capture butterfly diversity in both warm and cool seasons, as well as interannual variation [38,45]. A sampling period consisted of approximately one week where each of the ten transects was sampled three times. Transects were walked in random order from 0830 h to 1630 h (except if rained, in which case sampling was avoided). Each transect was patrolled continuously for 30 min, using a hand net to capture all observed butterflies at a radius of 5 m from the trail. All observed diurnal butterflies (Papilionoidea) were considered. Specimens were marked and released, and relevant information was also recorded, including time of day, transect and species name (when possible to identify, otherwise specimens were collected for further examination). All samplings were carried out by a single person (i.e., the first author). Collected specimens were stored and deposited in *Museo QCAZ Invertebrados* of *Pontificia Universidad Católica del Ecuador*. Taxonomic identification was confirmed by leading experts Keith Willmott (Nymphalidae, Riodinidae), Pierre Boyer (Hesperiidae), Jason Hall (Riodinidae) and Robert Busby (Lycaenidae). We followed [46] for further placement into subfamilies. It is noteworthy to mention that our sampling design precluded the possibility of obtaining records of species that are exclusively active at sunrise and sundown (e.g., crepuscular Satyrinae or nocturnal Hesperiidae).

### 2.4. Statistical Analyses

To compare temperature differences among seasons and among times of the day, we performed ANOVA analyses, using the Tukey method to test the significance of each comparison (95% confidence level). Median temperatures of cool vs. warm seasons, and of Morning vs. Midday vs. Afternoon were compared (see time-intervals classification below), using the Dplyr (v. 0.7.6) and Stats packages (v. 4.5) [47,48] in R (v. 4.5) [48].

For the seasonal analyses, we identified two intervals, consistent with monthly temperature fluctuations (Figure 3A). We obtained two intervals, namely “Warm” (August–January) and “Cool” (February–July) seasons. For the diel community analyses, we identified three time-intervals, consistent with daily temperature fluctuations (Figure 3C). To define precise time intervals, we divided the abundance dataset into intervals that had a similar number of samples. This allowed us to standardize sampling effort across the day (in terms of hours of sampling per community). We obtained intervals for “Morning” (0800 h to 1115 h), “Midday” (1116 h to 1300 h) and “Afternoon” (1301 h to 1630 h) assemblages.

To assess the adequacy of sampling efficiency of butterfly assemblages in our time intervals, we constructed interval-based rarefaction and extrapolation curves, using the iNEXT package (v. 3.0) [49] in R [48]. Rarefaction evaluates sampled diversity, and extrapolation evaluates estimated species diversity by standardizing sampling effort without losing information of species’ relative abundances [49,50,51,52]. 1000 bootstraps were set to obtain these curves.

We tested our hypothesis of seasonal and diel assembly partitioning by using three approaches. First, to visualize differences in the composition and structure of our communities, we performed a Non-metric multidimensional scaling ordination (NMDS) both at the species and family levels. This approach integrates empirical information on species presence (composition) and abundance (structure) within each sample. We used a Bray–Curtis similarity index after performing a Wisconsin double standardization of our data to equalize species contribution. This was supported by a permutational multivariate analysis of variance (PERMANOVA, 1000 permutations) to test the overall significance of the resulting patterns, and a SIMPER analysis to assess the contribution of each species to the observed differences. These analyses were performed using the Vegan package (v.2.5) [53] in R [48]. For the diel ordination, data of assemblages in similar times of the year (e.g., July 2015 + July 2018; October 2015 + October 2018; etc.) was combined to improve sampling coverage while reducing interannual variation [54].

Second, we numerically compared the communities’ diversities using “true” diversity metrics. Classic diversity indexes have been widely criticized, mainly for not holding a linear scale (thus complicating the interpretation of the magnitude of differences when comparing community diversities), for not accounting properly for sampling-effort differences, and for not providing insights on the biological significance of differences [50]. We therefore used Jost’s D, defined as the number of species in a community if all species were equally abundant [55]. In this framework, different values of a ‘q’ parameter incorporate sensitivity to species abundance, which allows intuitive comparisons. Hence, the diversity of order zero (q0) is completely insensitive to species abundances (and may be interpreted as richness). q1, weighs all species by their abundance, without favoring either common or rare species; and q2 gives more weight to abundant (or better sampled) species. These values may account for differential sampling efforts by standardizing by coverage, and confidence intervals may be used to assess the significance of differences. These analyses were performed using the iNEXT package [49] in R. To better estimate the richness of our three communities, we estimated the iChao2 measure [56] (10,000 bootstraps) using SpadeR (v. 1.2) [57] in R. Finally, we performed pairwise comparisons of butterfly assemblages. Because empirical similarity measures have a negative bias due to undetected species and undetected shared species in samples, we used estimated pairwise comparisons (1000 bootstraps) for q0, q1, and q2, using the SpadeR package in R [57].

Third, to effectively test the hypothesis of temporal niche partitioning and assess the relative contribution of underlying factors (abiotic or biotic) at the diel scale, we used an indirect method described by [58], which consist of estimating Pianka and Czechanowski overlap indexes and contrasting them with null-based models (see Figure 1). For this, we used the ROSARIO (Randomization of Sampled Activity with Replacement of Independent Observations) algorithm implemented in the TimeOverlap software (v. 1.0) [58], using 10,000 randomizations. This algorithm calculates the mean of the overlap indexes derived from all possible pairwise comparisons while generating means for randomly generated assemblages, maintaining much of the temporal structure of activity patterns of each species (thereby restricting generated patterns of activity to be biologically realistic). Significance is assessed comparing empirical values of overlap with the respective null distributions of values generated. Observed values may range from 0 (no overlap, or segregated activity. Figure 1A,B) to 1 (complete overlap, or aggregated activity. Figure 1C,D). A significantly low overlap value indicates temporally narrow, segregated activity, suggesting a high contribution of both abiotic and biotic interactions (Figure 1B); whereas a significantly high value indicates temporally narrow, aggregated activity, suggesting a relatively low relevance of interspecific interactions compared to abiotic factors (Figure 1D). No significant differences suggest that abiotic factors are not relevant for structuring temporal activity among species and thus their activity is widespread throughout time, and biotic factors may be relevant (species do not overlap, Figure 1A), or not (species overlap, Figure 1C). Data was plotted hourly to meet assumptions of the test.

## 3. Results

### 3.1. Weather Data

Mean environmental temperature was 27.4 and 28.9 °C for Cool and Warm seasons, respectively, and 26.8, 28.8 and 28.9 °C for Morning, Midday, and Afternoon intervals. Differences were of 1.5 °C for the Cool vs. Warm seasons (*p* < 0.001) and of 2.0, 2.1 and 0.04 °C for the Morning vs. Midday, Morning vs. Afternoon and Midday vs. Afternoon comparisons, respectively (*p* < 0.001, *p* < 0.001 and *p* = 0.01, respectively. Figure 3B,D). In other words, temperature differences between Cool and Warm seasons are similar to those between Morning and Midday, and Morning and Afternoon (Figure 3D).

### 3.2. Diversity Estimates

A total 1003 individuals of 222 species were recorded in 120 sampling hours (30 min. per transect × 240 total transects walked in the eight sampling periods), representing all families of diurnal Lepidoptera: Papilionidae, Hesperiidae, Pieridae, Nymphalidae, Riodinidae and Lycaenidae (Table S1). Empirical sampling completeness was >0.75 for all assemblages (morning, midday, afternoon, cool, warm), with curves showing stabilization and consistent patterns in extrapolated values, indicating adequate sampling effort (Figure S1).

**Figure 4:**
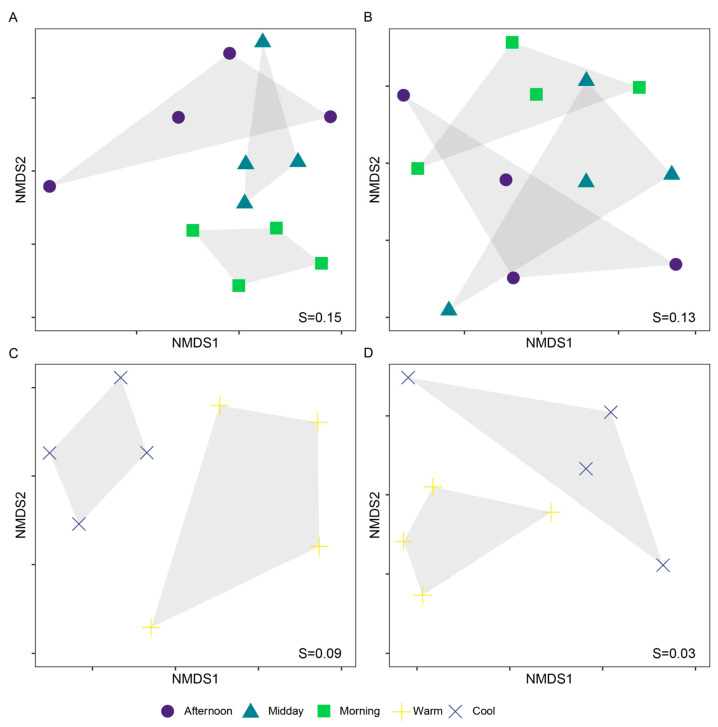
Bray–Curtis-based NMDS plots showing partitioning of butterfly assemblages in diel (**A**,**B**) and seasonal (**C**,**D**) scales, at the species (**A**,**C**) and family (**B**,**D**) levels. Differences between groups were significant for both seasonal and diel scales at the species level, but only significant for the seasonal scale at the family level (PERMANOVA. (**A**): R = 0.29, *p* < 0.001. (**B**): R = 0.23, *p* = 0.273. (**C**): R = 0.21, *p* < 0.001. (**D**): R = 0.46, *p* < 0.001). Stress (S) values are shown in each plot.

The differences between assemblages were significant for both seasonal and diel scales at the species level, but only significant for the seasonal scale at the family level (PERMANOVA. Diel, species: R = 0.29, *p* < 0.001. Diel, family: R = 0.21, *p* < 0.001. Seasonal, species: R = 0.23, *p* = 0.273. Seasonal, family: R = 0.46, *p* < 0.001). For the diel analysis, the species that contributed the most to discriminate assemblages were members of Danainae, Satyrinae and Riodinidae (SIMPER, Table S2). The results from PERMANOVA and pairwise comparisons consistently showed that Morning assemblages were more dissimilar to Afternoon than to Midday. Likewise, Midday assemblages were more similar to Afternoon than to Morning (Table 1, Figure 4). Likewise, Afternoon and Warm assemblages had more unique species compared to their respective counterpart assemblages; yet, for those assemblages (Morning & Midday, and Cool, respectively), 30% of their species were unique (Table 2).

**Figure 5:**
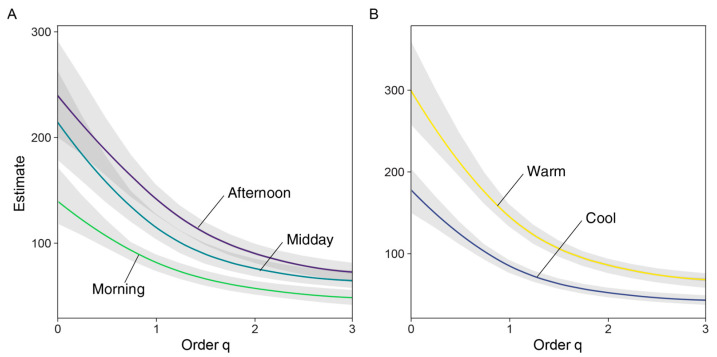
Estimated diversity profile plots (extrapolated at SC = 0.9), showing diel (**A**) and seasonal (**B**) assemblages’ diversity across orders of q. Light-colored polygons indicate SE for q values estimated from 1000 bootstrap replicates.

Results from the estimated diversity plot at a 0.9 Sampling Coverage (Figure 5) indicated that diversity across all orders of q was higher for Midday and Afternoon assemblages, compared to the Morning assemblage (Figure 4 and Figure 5). Likewise, the iChao2 estimate was higher for the Afternoon assemblage; this value was significantly higher compared to Morning, but not to Midday (Table 2).

### 3.3. Species Diel Activity and Niche Overlap

**Figure 6:**
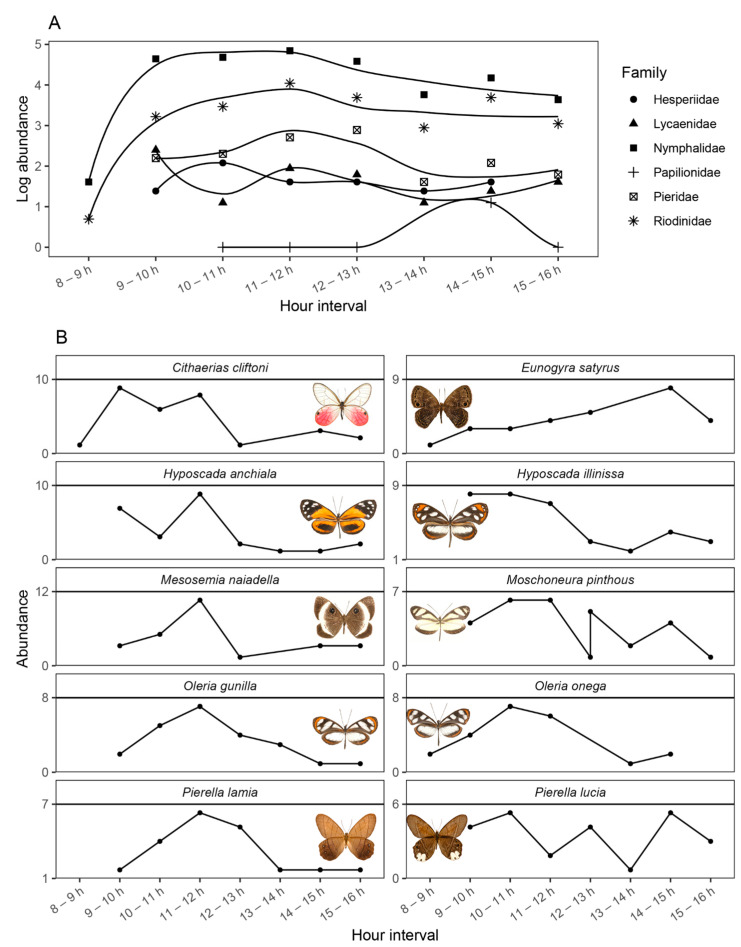
Diel activity patterns for Papilonoidea butterfly families (**A**) and the ten most common species (**B**) recorded in our study (summed over all samplings). Note that abundances in (**A**) were log-transformed to better visualize the activity patterns of families with low abundance records.

Results from the niche overlap analysis showed low overlap values, but significantly greater than expected by chance (Pianka = 0.29, *p* < 0.001; Czechanowski = 0.21, *p* < 0.001; 10,000 randomizations), indicating that, while there is a tendency for most species to be active towards a specific timeframe (i.e., near noon), there is a high turnover within activity hours. Our observations revealed that Nymphalidae and Riodinidae were the only families that showed activity in the early morning and peaked towards midday, whereas Papilionidae only began their activity near midday and peaked towards the afternoon. Pierdidae were also more active near noon, but their activity began after 0900 h, whereas Hesperiidae and Lycaenidae were similarly active throughout the day, also after 0900 h. The most abundant species recorded –members of Nymphalidae, Riodinidae, and Pieridae– showed contrasting patterns of activity. For example, whereas *Cithaerias cliftoni* was very active from the early morning to noon, *Eunogyra satirus* showed increased activity towards the 1400–1500 h interval. Two closely related Haeterini, *Pierella lamia* and *Pierella lucia*, showed opposite activity patterns, with the former aiming for midday and the latter avoiding such hours; whereas two closely related Ithomiini, *Oleria gunilla* and *Oleria onega*, showed coincident activity patterns, with an increase towards noon (Figure 6).

## 4. Discussion

Temporal activity patterns represent an important niche dimension that describes how species interact and exploit their environment [59,60]. While butterfly seasonal dynamics have been widely explored [8,9,10,11,38,40,54,61], our study demonstrates that biotic and abiotic patterns at diel scales are comparable in importance to those operating across seasons. We show that butterfly assemblages are not uniformly active throughout the day; rather, species exhibit distinct activity preferences. These findings highlight that diel partitioning should be considered alongside other coexistence mechanisms in hyperdiverse tropical systems.

Following niche partitioning theory, we expected butterfly assemblages to shift in their structure and composition while maintaining similar richness and abundance values across both diel and seasonal scales. Our results partially support this prediction: species composition and structure varied significantly across times of day and seasons. However, diversity was not evenly distributed. Both empirical and estimated curves showed higher diversity during warmer times of the day (Midday, Afternoon) and in the warm season, aligning instead with the predictions of temporal aggregation. This finding suggests that temperature availability is a key driver of butterfly assemblage structure across temporal scales. Temperature, nonetheless, exerts distinct pressures depending on scale. Seasonally, it structures forest microclimates and shapes species phenology, with butterflies often emerging in warmer periods after completing larval development on newly greening trees [7,8,39]; whereas diel temperature variation, in contrast, constrains metabolic activity in poikilotherms, thereby regulating butterfly flight activity [61,62].

Although temperature appears central, other abiotic and biotic factors likely interact with it to generate the patterns observed. For example, at a seasonal scale, studies have shown that most insects are highly abundant at the beginning of the rainy season [63,64]. For neotropical butterflies, richness tends to peak at the end of the rainy season [38,54], although [65] reported a tendency toward higher richness at the beginning of the dry season in a study on a seasonal floodplain in the Brazilian Amazon. Such studies also found that species composition did not differ between the rainy and dry seasons and suggested that seasonality might not be as strong an ecological filter in communities of low diversity composed of generalist species. In contrast, a butterfly-trapping study conducted at our study site by [8] showed significant species turnover throughout the year, with the highest species richness and abundance recorded in the months of high temperatures and intermediate precipitation. Similarly, a ten-year study at the northern edge of YNP showed strong species turnover between wet and dry seasons, but with maximum species diversity occurring during the dry seasons [38]. Our study provides evidence of strong seasonal turnover, with nearly half of the observed species being exclusive to the warm season. While clearly, even if significantly correlated [8], the effects of rainfall and those of temperature on the biology of butterflies cannot be conflated, our results certainly add to the hypothesis of environmental variables structuring seasonal partitioning of neotropical butterflies.

Previous research in butterflies and other insect groups often highlight the role of physiological constraints structuring diel activity. For instance, temperate butterflies frequently follow unimodal patterns peaking near midday, with activity largely constrained by thermal conditions. This has been interpreted as a compromise between avoiding avian predation and maximizing thermoregulatory efficiency [66]. Likewise, wasps in the Amazon and *Tirumala limniace* butterflies in Asia show bimodal activity centered on cooler morning and late afternoon hours, suggesting thermal avoidance more than competition [67,68]. Similarly, tabanid flies in a neotropical cloud forest exhibit a bimodal activity pattern influenced by morphology, temperature, and rainfall [69]. Yet, evidence also exists for endogenous timing and behavioral strategies promoting coexistence. The authors of [70] reported asynchronous patrolling among *Heliconius* butterflies of Costa Rica and Panamá, while [71] showed temporal segregation between Sphingidae and Saturniidae moths in French Guiana, linked to contrasting foraging strategies. In sum, these examples suggest that diel activity can reflect both ecological compromises and coexistence strategies, and further work is required to better understand its role in maintaining tropical hyperdiversity.

In our study, although many species converged in their activity around midday, their abundance peaks did not overlap, indicating that both abiotic constraints and biotic interactions may jointly structure diel patterns. However, while the effects of shifts in abiotic factors may be more generalizable across species, biotic interactions are much more complex to understand. For example, in a study similar to ours, [72] also found increasing activity of Brazilian skippers (Hesperiidae) towards midday, with contrasting activity patterns for congeneric species pairs. The authors suggested that diel partitioning may act as a reproductive isolation mechanism. We found strong evidence for diel community turnover with nearly 30% of unique species for each of our compared assemblages (Morning, Midday, and Afternoon); and further illustrated some contrasting hourly activity patterns both at the family level and for the most common species in our community. Yet it is noteworthy that while some closely related species showed opposite activity patterns, others showed coincident activity. Although our limited sampling size precludes the possibility of performing pairwise comparisons for multiple closely related species, this observation points to the effect of other interactions structuring assemblages. For example, while competition for a resource (e.g., nectar [29]) may promote temporal segregation, a pair of mimetic species may instead pursue a similar diel activity [63]. Thus, further research investigating diel activity and specific species interactions (whether among the most abundant tribes such as Haeterini or Ithomiini, among mimetic pairs or only nectarivores) will be useful to further elucidate the role of biotic interactions structuring coexistence in neotropical butterflies.

More general evolutionary perspectives across taxa may also shed light on diel activity patterns. It has been suggested that morphology and palatability mediate how species exploit thermal niches. For example, palatable, fast-flying butterflies, which require high metabolic inputs, tend to be active during the hottest hours of the day, while unpalatable, slow-flying taxa are more flexible [63]. Our results align with this view but add nuance: Satyrinae, often considered palatable, were dominant in the cool early morning (Data S1). This may be explained by their reliance on gliding flight, which is energetically efficient at low temperatures [33,42,73]. Species such as *Hermeuptychia hermes* are known to increase morning activity in cooler seasons [74], and Satyrinae more broadly have repeatedly colonized cold or shaded habitats, from rainforest understories to montane forests [75,76]. This highlights a possible link between flight morphology, thermal ecology, and evolutionary success.

It is important to acknowledge that diel activity patterns at the species level can influence detectability and, consequently, observed abundance patterns. Many tropical butterfly species exhibit vertical movements within the forest, with individuals shifting between understory and canopy strata throughout the day. Such behavior may reduce their likelihood of being detected during certain hours, potentially contributing to observed temporal fluctuations in community composition. Finally, the results of our study bear on climate change predictions. Rising temperatures may prompt heat-sensitive species to shift activity to earlier hours, while heat-tolerant species could expand activity periods. Yet such shifts may not guarantee persistence if species become desynchronized from hostplant phenology. Given ongoing biodiversity decline, particularly in insects [77,78], understanding how temporal activity mediates coexistence is crucial for forecasting the resilience of tropical communities.

## Figures and Tables

**Table 1:** Species shared and estimated pairwise similarity measures among subcommunities across different levels of q. Sampling error (SE) is shown in parentheses.

q Level	Similarity Measure	Diel			Seasonal
		Morning–Midday	Morning–Afternoon	Midday–Afternoon	Cool–Warm
-	Species shared	51	46	69	80
q0	Jaccard	0.43 (0.09)	0.34 (0.07)	0.62 (0.13)	0.37 (0.08)
q1	Horn	0.75 (0.05)	0.65 (0.05)	0.74 (0.07)	0.79 (0.05)
q2	Morisita–Horn	0.43 (0.12)	0.35 (0.07)	0.62 (0.13)	0.82 (0.05)

**Table 2:** Species richness (s), abundances (n), sampling coverage (SC) and iChao2 estimator for the three diel assemblages and the two seasonal assemblages.

	Diel			Seasonal	
	Morning	Midday	Afternoon	Cool	Warm
n	309	390	304	381	589
s observed	93	127	129	109	180
s unique	33	42	50	29	100
SC	0.87	0.84	0.77	0.86	0.85
iChao2 (SE 95%)	152 (12)	231 (23)	258 (28)	184 (19)	330 (24)

## Data Availability

The original data presented in the study are openly available in GitHub at https://github.com/sebas-menag/temppart (accessed on 20 October 2025).

## Supplementary Materials

The following supporting information can be downloaded at: https://www.mdpi.com/article/10.3390/insects16121247/s1, Figure S1: Sampling effort metrics; Table S1: Species richness and abundance in YFDP at morning, midday and afternoon in the years 2015, 2016 and 2018; Table S2: Results from the SIMPER analysis. Data S1: Family observed abundances for the morning, midday, and afternoon assemblages.

## Abbreviations

The following abbreviations are used in this manuscript:

YNP	Yasuní National Park
ECY	Estación Científica Yasuní
YFDP	Yasuní Forest Dynamics Plot
