# Supplementary material for: Diel Versus Seasonal Butterfly Community Partitioning in a Hyperdiverse Tropical Rainforest
Source: Insects. 2025 Dec 10;16(12):1247. doi: 10.3390/insects16121247 (PMC12733456; doi:10.3390/insects16121247)
Supplement: Supplementary file 1 [file insects-16-01247-s001.zip › insects-3923841-supplementary.pdf]

Supplementary Material

Diel Versus Seasonal Butterfly Community Partitioning in a Hyperdiverse Tropical Rainforest

Sebastián Mena [1,2,*], Janeth Rentería [1] and María F. Checa [1,*]

[1] WasiLab—Laboratorio de Estudios en Sostenibilidad, Pontificia Universidad Católica del Ecuador, Quito 170525, Ecuador
[2] Museo de Zoología QCAZ Invertebrados, Escuela de Ciencias Biológicas, Pontificia Universidad Católica del Ecuador, Quito 170525, Ecuador
* Correspondence: dsmenag@gmail.com (S.M.); mfcheca@puce.edu.ec (M.F.C.)

Figure S1. Sampling effort metrics. A. Completeness curve. B. Sample-sized-based sampling curve. C. Coverage-based sampling curve.

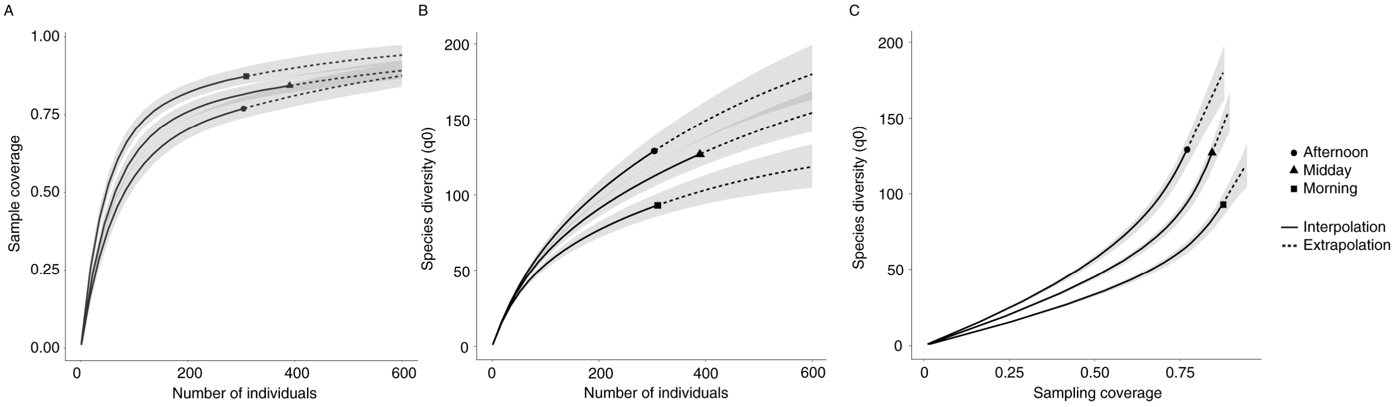

Table S1. Species richness and abundance in YFDP at morning, midday and afternoon in the years 2015, 2016 and 2018.

month	jul15+ jul18	oct15+ oct18	feb16+ jan18	may16 + may18	jul15+ jul18	oct15 + oct18	feb16 + jan18	may16 + may18	jul15+ jul18	oct15+ oct18	feb16+ jan18	may16+ may18
time	morning	morning	morning	morning	midday	midday	midday	midday	afternoon	afternoon	afternoon	afternoon
Adelpha cocala	0	1	0	0	0	3	0	0	0	0	0	0
Aeria eurimeda	0	0	0	0	0	0	0	0	0	0	1	1
Alesa amesis	0	0	0	0	0	0	0	0	0	1	0	0
Aguna coelus	0	0	0	0	0	1	0	0	0	0	0	0
Amarynthis meneria	0	0	0	0	0	2	0	1	0	0	0	0
Amiga arnaca	0	0	2	1	1	0	0	0	0	2	0	0
Arawacus seperata	0	0	1	0	0	1	0	0	0	0	1	0
Anastrus tolimus	0	2	0	0	0	0	0	0	0	0	0	0
Anteros stramentarius	1	1	0	0	0	0	0	0	0	0	0	0
Antirrhea philoctetes	0	0	1	0	0	0	0	0	0	0	0	0
Arcas imperialis	0	0	0	0	0	1	0	0	0	0	0	0
Asterope markii	0	0	0	0	0	0	0	0	0	1	0	0
Astraptes fulgerator	0	1	0	0	0	1	0	0	1	0	0	0
Astraptes janeira	0	0	0	0	0	1	0	0	0	0	0	0
Autochton zarex	0	1	0	0	0	0	0	0	0	0	1	0
Bia actorion	0	0	5	1	1	0	0	1	3	1	0	1
Brevianta ematheon	0	2	0	0	0	0	0	0	0	0	0	0
Brevioleria arzalia	2	2	0	1	2	4	1	2	2	4	0	0
Cabirus procas	1	1	1	0	0	0	0	1	0	0	0	0
Callimormus sp1	0	0	0	1	0	0	0	0	0	0	0	0
Calycopis sp.1	1	1	0	0	0	0	0	0	0	0	0	0
Caligo idomeneus	0	0	0	0	0	0	0	0	0	0	0	0
Callithomia lenea	0	0	0	0	0	0	1	0	0	1	0	0
Calycopis sp.2	2	2	1	0	1	1	2	0	0	0	3	1
Cartea vitula	0	0	0	0	0	1	0	0	0	0	0	0
Celaenorrhinus autochton	0	0	0	0	0	0	0	0	0	1	0	0
Celaenorrhinus syllius	2	1	0	0	0	0	0	2	0	1	1	0
Celmia celmus	1	3	0	0	0	2	0	0	0	2	0	0
Caria sponsa	0	0	0	0	0	0	0	0	0	1	0	0
Cartea vitula	0	0	0	0	0	0	0	0	0	1	0	0
Charis anius	0	0	0	0	1	0	1	0	0	0	0	0
Catoblepia xanthicles	0	0	0	0	0	0	0	0	0	1	0	0
Chloreuptychia agatha	0	0	2	0	0	1	0	0	0	0	0	1
Chloreuptychia herseis	1	0	1	1	2	0	1	2	0	0	2	0
Cithaerias cliftoni	6	4	2	5	3	1	3	2	3	1	0	1
Cithaerias pireta	2	1	0	0	0	0	0	0	0	0	0	0
Crocozona coecias	0	0	0	0	0	2	0	0	0	0	0	0
Cynea hicsos	0	0	0	0	0	0	1	0	0	0	0	0
Colobura dirce	0	0	0	0	0	0	0	0	0	1	0	0
Detritivora matic	0	0	0	0	1	0	2	1	0	0	0	1
Dismorphia laja	0	0	0	0	1	0	0	0	0	0	0	0

Dismorphia theucharila	0	0	0	2	2	3	1	1	0	2	0	0
Dynamine artemisa	0	1	0	0	0	4	1	0	0	0	1	0
Dynamine chryseis	0	0	0	0	0	0	1	0	0	0	1	0
Dynamine sosthenes	0	0	0	0	0	1	0	0	0	0	1	0
Echydna punctata	0	0	0	0	0	1	1	0	5	0	0	0
Emesis ocypore	0	0	0	0	0	1	0	0	0	0	0	0
Emesis spreta	0	0	0	0	0	0	0	1	0	0	0	1
Eresia pelonia	0	0	0	0	0	0	1	0	0	2	0	0
Eunica amelia	0	0	0	0	0	1	0	0	0	1	0	0
Entheus gentius	0	0	0	0	0	0	0	0	1	0	0	0
Eunica concordia	0	1	0	0	0	0	0	0	0	0	0	0
Eunogyra satyrus	4	4	0	1	1	3	3	3	3	2	2	5
Euptychia picea	0	1	0	0	1	1	3	1	0	0	0	0
Eurema albula	0	1	0	0	0	1	0	0	0	0	0	0
Euptychia fernandae	0	0	0	0	0	0	0	0	0	1	0	0
Eurybia halimede	0	0	0	2	0	1	0	0	0	0	0	0
Eurybia latifasciata	0	0	0	1	5	0	0	0	0	0	0	0
Euptychia picea	0	0	0	0	0	0	0	0	0	1	1	0
Euptychia jesia	0	0	0	0	0	0	0	0	1	0	0	0
Eurybia leucolopha	0	0	0	1	1	2	0	2	4	0	0	3
Eurybia nr.fulgens	0	0	1	1	0	0	0	0	0	1	1	0
Eurybia nr.lamia	0	0	0	0	1	0	0	1	0	0	0	0
Eurybia nr.nicaeus	0	0	0	0	0	1	5	0	0	1	1	1
Euryphellus euribates	0	0	1	0	0	0	0	0	0	0	0	0
Eurybia sp5	1	2	1	2	1	0	0	1	1	3	1	0
Euselasia alcmena	0	0	0	0	0	2	1	0	2	0	0	0
Euselasia lysimachus	0	1	0	0	0	0	0	0	0	2	0	0
Euselasia effima	0	0	0	0	0	1	0	0	0	0	0	0
Euselasia erilis	0	0	0	0	1	0	0	0	0	0	0	0
Euselasia janigena	0	0	0	0	1	0	0	0	0	0	0	0
Euselasia nr.cyanofusa	0	0	0	0	0	0	1	0	0	0	0	0
Euselasia uria	0	0	0	0	0	1	0	0	0	0	0	0
Euselasia nauca	0	0	0	0	0	0	0	0	0	1	0	0
Exorbaetta metanira	0	0	0	0	0	0	0	0	0	1	0	0
Forbestra olivencia	2	1	0	0	0	0	0	0	0	1	0	0
Godyris zavaleta	0	0	0	1	0	0	0	0	0	0	0	0
Exoplisia cadmeis	0	0	0	0	0	2	0	0	0	0	0	0
Forbestra olivencia	0	0	0	0	1	4	1	0	0	0	0	0
Glutophrissa drusilla	0	0	0	0	0	2	0	0	1	0	0	0
Godyris zavaleta	0	0	0	0	1	1	0	0	1	0	1	1
Haetera piera	4	2	2	2	1	1	0	0	3	1	0	1
Heliconius aeode	0	1	0	0	0	2	0	0	0	3	0	0
Heliconius elevatus	0	0	0	0	1	1	2	0	0	0	0	0
Heliconius erato	0	0	0	0	1	1	0	0	0	1	0	0
Heliconius hecale	0	0	0	0	0	0	0	0	0	0	0	1
Heliconius metharme	0	0	0	0	0	0	0	0	0	1	0	0
Heliconius numata	0	1	0	0	1	2	0	0	1	1	0	5
Hypoleria sarepta	0	0	1	1	0	0	0	0	0	0	0	0

Hyaliris sp.1	0	0	0	0	0	1	0	0	0	0	0	0
Heraclides torquatus	0	0	0	0	0	0	0	0	0	1	0	0
Hyposcada anchiala	4	3	3	2	1	2	7	2	0	0	0	0
Hermeuptychia sp1	0	0	0	0	0	0	0	0	0	0	2	0
Hyphilaria parthenis	0	0	0	0	0	0	0	0	0	0	1	0
Hyposcada illinissa	2	0	4	3	3	4	5	2	4	4	0	2
Hyposcada kena	2	1	3	0	1	3	0	2	0	0	0	0
Hyposcada anchiala	0	0	0	0	0	0	0	0	3	1	0	0
Hypothyris anastasia	0	0	3	0	3	2	1	0	0	0	0	0
Hypothyris fluonia	1	2	2	0	1	4	3	0	0	0	0	0
Hyposcada kena	0	0	0	0	0	0	0	0	1	0	0	0
Hypostrymon asa	0	0	0	0	0	0	0	0	0	1	0	0
Hypothyris anastasia	0	0	0	0	0	0	0	0	1	3	0	0
Hypothyris fluonia	0	0	0	0	0	0	0	0	1	3	2	0
Itaballia demophile	0	0	0	0	0	3	1	0	2	3	0	0
Itaballia pandosia	1	2	0	0	0	6	1	0	0	1	0	1
Ithomeis aurantiaca	0	1	0	0	0	0	0	0	0	0	2	0
Ithomia amarilla	2	2	1	2	5	3	1	2	1	2	0	2
Ithomia salapia	0	0	0	0	1	0	0	0	0	0	0	0
Janthecla rocena	0	0	0	0	0	0	0	0	0	1	0	0
Janthecla sista	2	2	0	0	0	3	0	0	0	0	1	0
Leucidia brephos	1	2	0	0	0	0	0	0	0	0	0	0
Leucochimona icare	0	1	0	0	0	0	0	0	0	0	0	0
Livendula jasonhalli	0	0	0	0	0	0	1	0	0	1	0	0
Leucidia brephos	0	0	0	0	0	0	0	0	0	1	0	0
Livendula amasis	0	0	0	0	0	0	0	0	0	0	1	0
Livendula violacea	0	0	0	0	0	1	0	0	0	0	0	0
Magneuptychia analis	0	0	0	0	0	0	1	0	0	0	0	1
Magneuptychia agatha	1	1	0	0	0	0	0	0	0	0	0	0
Magneuptychia libye	0	0	0	0	0	1	0	0	0	0	0	0
Magneuptychia iris	0	0	1	0	0	0	0	0	0	0	0	0
Magneuptychia ocypete	0	0	1	0	1	1	2	1	0	1	0	1
Magneuptychia sp.2	1	4	2	1	1	1	2	1	1	1	3	3
Magneuptychia pax	1	1	0	0	0	0	0	0	0	0	1	0
Magneuptychia sp.3	0	0	0	0	0	0	1	0	0	0	0	0
Magneuptychia sp.a	0	0	0	0	0	1	1	0	0	1	0	0
Magneuptychia tricolor	0	1	1	0	0	1	2	1	0	1	0	2
Melinaea mothone	0	0	0	0	0	1	0	0	0	0	0	0
Marela tamyris	0	0	0	0	0	0	0	0	0	0	1	0
Mechanitis messenoides	0	0	0	0	1	0	0	0	0	2	0	0
Mesosemia cippus	0	0	0	0	1	0	0	0	0	0	0	0
Mechanitis mazaeus	1	0	0	0	0	0	0	0	0	0	0	0
Mesophthalma idotea	0	0	0	1	0	0	0	0	0	0	0	0
Mesosemia amarantus	0	0	0	0	0	0	0	0	0	0	1	0
Mesosemia erinnya	0	0	1	0	0	0	0	0	0	0	0	0
Mesosemia eumene	0	0	2	2	1	0	0	1	0	0	0	0
Mesosemia macella	0	0	0	0	0	0	0	1	0	0	0	0
Mesosemia machaera	0	0	1	0	0	0	0	0	0	0	0	0

Mesosemia messeis	0	0	0	0	1	0	0	0	1	2	0	0
Mesosemia metura	0	0	0	0	0	0	1	1	0	0	1	0
Mesosemia naiadella	1	1	7	0	0	2	8	2	1	2	2	1
Minotauros lampros	1	1	0	0	0	0	0	0	0	0	0	0
Mesosemia jeziela	0	0	0	0	0	0	0	0	0	1	0	1
Mesosemia judicialis	0	0	0	0	0	0	0	0	0	1	0	1
Mesosemia thymetus	0	0	0	0	0	0	0	1	1	1	0	0
Mesosemia minos	0	0	0	0	0	0	0	0	0	0	1	0
Mesosemia misipsa	0	0	0	0	0	0	0	0	0	0	0	1
Metacharis regalis	0	0	0	0	0	0	0	0	0	0	1	0
Morpho deidamia	0	0	1	1	0	0	0	0	0	0	0	0
Methionopsis ina	0	0	0	0	0	0	0	0	1	0	0	0
Methona curvifascia	0	0	0	0	0	0	0	0	0	2	2	0
Methone cecilia	0	0	0	0	0	0	0	0	0	1	0	0
Metacharis lucius	0	0	1	0	0	0	0	0	0	0	0	0
Methona curvifascia	0	0	0	1	0	0	0	0	0	0	0	0
Minotauros lampros	0	0	1	0	0	0	0	0	0	0	0	0
Morpho helenor	2	1	0	1	1	0	0	0	0	0	0	0
Moschoneura pinthous	0	4	3	0	2	5	4	2	1	4	1	3
Napaea heteroea	1	0	0	0	1	1	0	0	0	0	0	0
Napeogenes pharo	0	0	0	0	0	1	0	0	0	0	0	0
Napeogenes achaea	0	0	0	0	0	0	0	0	0	0	1	0
Napeogenes rhezia	0	0	0	0	0	1	0	0	0	1	0	0
Napeogenes sylphis	0	0	0	0	0	1	2	0	1	1	0	0
Nessaea hewitsonii	0	1	0	1	0	0	0	0	0	0	0	0
Nymphidium acherois	0	0	0	0	0	0	1	0	0	0	0	0
Nessaea obrinus	0	0	0	0	0	0	0	0	1	0	0	0
Nymphidium cachrus	0	0	1	0	0	0	0	0	0	0	0	1
Nymphidium lisimon	4	4	0	1	0	2	3	0	1	0	0	0
Nymphidium medusa	0	0	0	0	0	1	0	0	0	2	1	0
Nymphidium plinthobaphis	1	1	1	0	0	0	0	0	0	0	0	0
Oleria agarista	0	0	0	0	1	0	0	0	0	0	0	0
Oleria assimilis	0	0	0	0	1	1	0	0	1	0	0	0
Oleria gunilla	1	1	2	2	0	0	0	0	2	3	0	0
Oleria gunilla	0	0	0	0	4	2	2	3	0	0	0	0
Oleria ilerda	0	0	0	0	0	0	1	1	0	0	0	0
Oleria onega	1	3	1	6	1	1	4	3	0	2	1	0
Oleria sexmaculata	1	0	2	3	1	2	4	0	1	2	0	1
Ostrinotes tympania	0	0	0	0	0	0	1	0	0	0	0	0
Parides aeneas	0	0	0	0	0	1	0	0	2	1	0	0
Parides chabrias	0	0	1	0	0	0	0	0	0	0	0	0
Panacea procilla	0	0	0	0	0	0	0	0	0	1	0	0
Parvospila emylius	0	1	0	0	0	1	0	0	0	1	0	0
Phareas coeleste	0	0	1	0	0	0	0	0	0	0	0	0
Perrhybris pamela	0	0	0	0	0	0	0	0	1	0	0	0
Parides lysander	0	0	0	0	0	1	0	0	0	0	0	0
Pierella hortona	5	2	1	0	0	0	0	0	1	1	0	1
Pierella lamia	5	1	1	0	3	0	4	3	3	1	1	2

Pierella lena	3	2	4	1	1	0	4	2	1	2	0	0
Pierella lucia	4	0	4	0	2	1	1	3	7	1	1	1
Pierella hortona	0	0	0	0	2	2	0	0	0	0	0	0
Pseudonascus paullinae	0	0	0	0	0	0	0	0	1	0	0	0
Pseudoscada florula	0	2	2	2	1	1	2	2	1	1	1	1
Posttaygetis penelea	0	0	0	0	0	0	0	1	0	0	0	0
Pteronymia primula	0	0	0	0	1	1	1	0	0	0	0	0
Pyrrhogyra otolais	0	0	0	0	0	3	0	0	1	0	0	0
Rhetus periander	0	0	0	0	0	0	0	0	0	1	0	0
Quadrus fanda	0	0	0	0	0	1	0	0	0	0	0	0
Saliana salius	0	0	0	0	0	0	1	0	0	0	0	0
Sarota completa	0	0	0	0	0	0	0	1	0	0	0	0
Semomesia croesus	0	0	1	0	0	0	0	0	0	0	0	0
Stalachtis calliope	1	1	0	0	0	0	1	0	0	1	1	0
Scada zibia	0	0	0	0	2	0	0	0	0	0	0	1
Siderus sp.	0	0	0	0	0	0	0	0	0	0	1	0
Stalachtis euterpe	0	0	0	0	0	0	0	0	1	3	0	0
Sostrata pusilla	0	0	0	0	0	1	0	0	0	0	0	0
Splendeuptychia sp1	0	0	0	0	0	1	0	0	0	0	0	0
Stalachtis phaedusa	0	0	0	0	0	1	0	0	0	1	0	0
Strephonota sp.	0	1	0	0	0	0	0	1	0	0	0	0
Symmachia phaedra	0	1	0	0	0	0	0	0	0	0	0	0
Synargis abaris	0	0	0	0	0	0	0	0	0	1	0	0
Tarsoctenus coritus	0	0	0	0	0	0	0	0	0	1	0	0
Taygetina sp1	0	0	0	0	0	0	0	0	0	0	0	1
Taygetis cleopatra	0	0	0	0	0	0	0	0	0	0	0	1
Taygetis sosis	0	0	0	0	0	0	1	0	1	0	0	0
Taygetina sp1	0	0	0	0	0	0	0	0	0	0	0	0
Theope sobrina	0	0	0	0	0	0	0	0	0	2	0	0
Tigridia acesta	0	0	0	0	0	0	0	0	0	0	1	0
Taygetis sosis	1	0	0	0	0	0	0	0	0	0	0	0
Taygetis sp2	0	1	0	0	0	0	0	0	0	0	0	0
Vila emilia	0	0	0	0	0	5	0	0	0	2	1	1
Terenthina terentina	0	0	0	0	0	1	0	0	0	0	0	0
Theope pedias	0	0	0	0	0	0	0	1	0	0	0	0
Theope philotes	0	0	0	0	0	1	0	0	0	0	1	0
Taygetis thamyra	0	0	1	0	0	0	0	0	0	0	0	0

Table S2. Results from the SIMPER analysis.

$morning_midday

Mesosemia_naiadella	Oleria_gunilla.1	Pierella_lamia	Cithaerias_cliftoni
0.02788222	0.05405034	0.07510378	0.09552859
Moschoneura_pinthous	Haetera_piera	Pierella_lucia	Nymphidium_lisimon
0.11571141	0.13423444	0.15259647	0.17083748
Oleria_onega	Hyposcada_anchiala	Pierella_hortona	Eunogyra_satyrus
0.18882269	0.20653822	0.22406195	0.2399798
Hyposcada_illinissa	Itaballia_pandosia	Oleria_gunilla	Pierella_lena
0.25498901	0.26960594	0.28363004	0.2976509
Oleria_sexmaculata	Hypothyris_fluonia	Eurybia_latifasciata	Hypothyris_anastasia
0.31161419	0.32553323	0.33926217	0.35269301
Bia_actorion	Dismorphia_theucharila	Ithomia_amarilla	Eurybia_nr.nicaeus
0.36599303	0.3787651	0.3913417	0.40359039
Forbestra_olivencia.1	Euptychia_picea	Hyposcada_kena	Janthecla_sista
0.41526623	0.42653271	0.43778795	0.44835517
Brevioleria_arzalia	Eurybia_leucolopha	Celmia_celmus	Detritivora_matic
0.45883372	0.46902336	0.47856379	0.48806675
Dynamine_artemisa	Mesosemia_eumene	Magneuptychia_ocypete	Vila_emilia
0.49753715	0.50689158	0.51596371	0.52480326
Magneuptychia_sp.2	Eurybia_sp5	Pierella_hortona.1	Heliconius_elevatus
0.53341973	0.54203499	0.5505869	0.55905517
Chloreuptychia_herseis	Calycopis_sp.2	Celaenorrhinus_syllius	Morpho_helenor
0.56740605	0.57562644	0.58382151	0.59171282
Itaballia_demophile	Amiga_arnaca	Magneuptychia_tricolor	Pseudoscada_florula
0.59911271	0.60600834	0.61281235	0.61945882
Adelpha_cocala	Cithaerias_pireta	Forbestra_olivencia	Nymphidium_plinthobaphis
0.62603488	0.63258498	0.63913509	0.64565915
Leucidia_brephos	Pteronymia_primula	Amarynthis_meneria	Heliconius_numata
0.65208503	0.65845714	0.66479556	0.67096989
Eurybia_halimede	Napeogenes_sylphis	Euselasia_alcmena	Cabirus_procas
0.67705452	0.68301474	0.68864672	0.69403176
Eurybia_nr.lamia	Chloreuptychia_agatha		
0.69934241	0.70465014		

$morning_afternoon

Cithaerias_cliftoni	Hyposcada_anchiala	Pierella_lucia	Oleria_onega
0.03025562	0.05791501	0.08330641	0.10625279
Eunogyra_satyrus	Nymphidium_lisimon	Mesosemia_naiadella	Pierella_lena
0.12545915	0.14459696	0.16310537	0.1810495
Moschoneura_pinthous	Eurybia_leucolopha	Heliconius_numata	Hyposcada_illinissa
0.19847761	0.21534287	0.23180265	0.24813338
Bia_actorion	Pierella_lamia	Haetera_piera	Pierella_hortona
0.2640047	0.27953909	0.2950583	0.3100783
Hyposcada_kena	Brevioleria_arzalia	Hypothyris_fluonia.1	Magneuptychia_sp.2
0.32343192	0.33664806	0.34960378	0.36160385
Oleria_gunilla	Calycopis_sp.2	Oleria_sexmaculata	Echydna_punctata

0.37352121	0.3852763	0.39657199	0.40782118
Hypothyris_fluonia	Mesosemia_eumene	Itaballia_demophile	Celmia_celmus
0.4187986	0.42879994	0.43862081	0.44828523
Morpho_helenor	Pseudoscada_florula	Eurybia_sp5	Janthecla_sista
0.45770232	0.46708951	0.47637198	0.48534336
Methona_curvifascia	Vila_emilia	Hyposcada_anchiala.1	Chloreuptychia_herseis
0.49427551	0.50318659	0.51170983	0.52017685
Amiga_arnaca	Godyris_zavaleta.1	Hypothyris_anastasia.1	Stalachtis_euterpe
0.52824471	0.53585817	0.5434292	0.55100023
Ithomia_amarilla	Magneuptychia_tricolor	Eurybia_nr.nicaeus	Dismorphia_theucharila
0.55852099	0.56585518	0.57299253	0.58007148
Celaenorrhinus_syllius	Itaballia_pandosia	Forbestra_olivencia	Hypothyris_anastasia
0.58700441	0.59379225	0.60052684	0.60722974
Cithaerias_pireta	Cabirus_procas	Nymphidium_plinthobaphis	Heliconius_aeode
0.61388046	0.62050127	0.62712209	0.63374128
Leucidia_brephos	Ithomeis_aurantiaca	Parides_aeneas	Nymphidium_medusa
0.64025011	0.64655496	0.65282837	0.65906817
Chloreuptychia_agatha	Mesosemia_messeis	Eurybia_halimede	Hermeuptychia_sp1
0.66494448	0.67074178	0.67627452	0.68165921
Aeria_eurimeda	Hypoleria_sarepta	Morpho_deidamia	Nessaea_hewitsonii
0.68702283	0.69202349	0.69702416	0.70191285

$midday_afternoon

Hyposcada_anchiala	Oleria_gunilla.1	Mesosemia_naiadella	Pierella_lucia	Ithomia_amarilla
0.0251342	0.05020021	0.06905819	0.085823	0.1017209
Oleria_onega	Hyposcada_illinissa	Eurybia_leucolopha	Heliconius_numata	Hypothyris_fluonia
0.11760904	0.13335071	0.14871523	0.16387415	0.17897256
Brevioleria_arzalia	Moschoneura_pinthous	Pierella_lamia	Vila_emilia	Pierella_lena
0.19403676	0.20853394	0.22226386	0.23578089	0.24917903
Dismorphia_theucharila	Hyposcada_kena	Euptychia_picea	Eurybia_nr.nicaeus	Itaballia_pandosia
0.26210901	0.2749433	0.28771442	0.30047899	0.31318799
Cithaerias_cliftoni	Hypothyris_anastasia	Echydna_punctata	Hypothyris_fluonia.1	Eurybia_latifasciata
0.32587889	0.33844133	0.35089898	0.36308835	0.37511139
Eunogyra_satyrus	Oleria_sexmaculata	Forbestra_olivencia.1	Itaballia_demophile	Chloreuptychia_herseis
0.38676893	0.39827413	0.40937941	0.42025289	0.43044241
Calycopis_sp.2	Magneuptychia_sp.2	Nymphidium_lisimon	Oleria_gunilla	Dynamine_artemisa
0.44053453	0.45023354	0.45990237	0.46922175	0.47837188
Haetera_piera	Methona_curvifascia	Bia_actorion	Pierella_hortona.1	Heliconius_elevatus
0.48710502	0.49548309	0.50383413	0.5119954	0.52006916
Hyposcada_anchiala.1	Magneuptychia_tricolor	Detritivora_matic	Eurybia_sp5	Hypothyris_anastasia.1
0.52810944	0.53594979	0.54349298	0.55100248	0.55820737
Stalachtis_euterpe	Heliconius_aeode	Celaenorrhinus_syllius	Euselasia_alcmena	Magneuptychia_ocypete
0.56541225	0.57247425	0.57950308	0.58647442	0.59307771
Janthecla_sista	Pyrrhogyra_otolais	Pierella_hortona	Napeogenes_sylphis	Parides_aeneas
0.5996742	0.60599792	0.61228368	0.61849288	0.62458561
Pteronymia_primula	Amarynthis_meneria	Nymphidium_medusa	Scada_zibia	Mesosemia_messeis
0.6306628	0.63671566	0.64266346	0.64858238	0.65442581
Celmia_celmus	Eurybia_nr.lamia	Mesosemia_eumene	Adelpha_cocala	Hermeuptychia_sp1

0.65979101	0.66489642	0.67000183	0.67502991	0.68001439
Ithomeis_aurantiaca	Aeria_eurimeda	Amiga_arnaca	Mechanitis_messenoides	Oleria_ilerda
0.68499887	0.68996558	0.69484932	0.69973306	0.70443043

Data S1. Family observed abundances for the morning, midday, and afternoon assemblages. Nymphalidae is divided in subfamilies. Cladogram after Espeland et al. (2018).

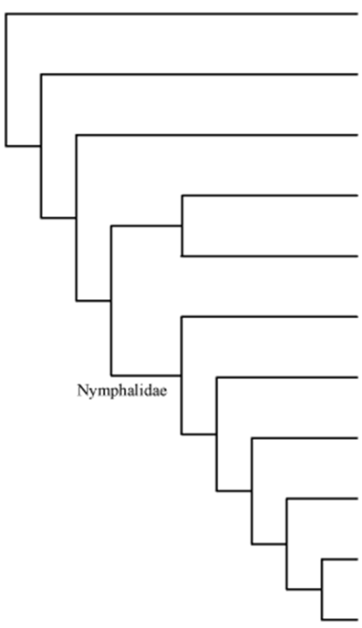

Subfamily	Morning	Midday	Afternoon	Total
Papilionidae	1	2	4	7
Hesperiidae	13	9	10	32
Pieridae	16	35	21	72
Riodinidae	64	93	88	245
Lycaenidae	19	14	12	45
Danainae	85	129	71	285
Limenitidinae	1	3	0	4
Heliconiinae	2	11	13	26
Biblidinae	4	16	12	32
Nymphalinae	0	1	4	5
Satyrinae	104	77	69	250
